# Genome-Wide Characterization of the *ScLOR* Gene Family in Wild Tomato *Solanum lycopersicoides* Reveals *ScLOR16* as a Negative Regulator of Cold-and Drought-Stress Tolerance

**DOI:** 10.3390/biology15171542

**Published:** 2026-09-04

**Authors:** Xinyi Tang, Yueqi Huang, Aoxue Wang, Liguo Zhang, Mingfang Feng

**Affiliations:** 1 College of Life Sciences, Northeast Agricultural University, Harbin 150030, China; 2 Institute of Industrial Crops, Heilongjiang Academy of Agricultural Sciences, Harbin 150086, China; 3 College of Horticulture, Northeast Agricultural University, Harbin 150030, China

**Keywords:** LOR gene family, *Solanum lycopersicoides*, *ScLOR16*, cold stress, drought stress, virus-induced gene silencing (VIGS), antioxidant defense

## Abstract

The LOR (*LURP*-one related) gene family encodes proteins containing conserved LOR domains; however, its functions in plant abiotic stress responses remain largely unexplored. In this study, we systematically identified and characterized the LOR gene family in the stress-tolerant wild tomato *Solanum lycopersicoides* using comprehensive bioinformatic analyses and conducted functional validation of the candidate gene *ScLOR16*. A total of 19 *ScLOR* members were identified and classified into eight phylogenetic subgroups. Numerous cis-acting elements associated with responses to abscisic acid (ABA), cold, and drought, including ABRE, LTR, and MBS, were detected in the promoter regions, suggesting that the *ScLOR* family may be broadly involved in ABA-mediated stress signaling pathways. RT-qPCR analysis revealed that *ScLOR16* expression was significantly induced by both cold and drought treatments. Subcellular localization assays demonstrated that ScLOR16 is localized in both the nucleus and cytoplasm. Virus-induced gene silencing (VIGS) was subsequently employed to generate *ScLOR16*-silenced plants. Following 24 h of cold treatment at 4 °C and four days of drought stress, *ScLOR16*-silenced seedlings exhibited significantly less severe wilting symptoms than empty-vector controls. Physiological analyses showed that silenced plants exhibited enhanced superoxide dismutase (SOD) and peroxidase (POD) activities, increased proline accumulation, and decreased thiobarbituric acid-reactive substances (TBARS) content. Collectively, these results indicate that reduced *ScLOR16* transcript levels are associated with enhanced cold and drought tolerance, accompanied by alterations in antioxidant defense and osmoprotection-related physiological markers. This study yields new insights into the evolution and stress-related functions of the *ScLOR* family.

## Simple Summary

The LOR gene family’s abiotic stress functions are largely unknown in wild tomato. We identified 19 *ScLOR* genes from stress-tolerant *Solanum lycopersicoides* and performed comprehensive bioinformatic analyses. Their promoters contain abundant ABA, cold and drought responsive cis-elements. RT-qPCR revealed *ScLOR16* was sharply induced by cold and drought. Subcellular localization showed ScLOR16 localizes in the nucleus and cytoplasm. VIGS of *ScLOR16* alleviated leaf wilting under cold and drought. Silenced plants had higher SOD/POD activity, more proline and lower TBARS, indicating stronger antioxidant and osmotic protection. Overall, *ScLOR16* is likely involved in the modulation of cold and drought stress responses. This work characterizes evolutionary features of the LOR family and provides basic insights for studying stress-adaptation mechanisms in *Solanum lycopersicoides*.

## 1. Introduction

Plants frequently encounter various abiotic stresses, including low temperature, drought, salinity, heavy metal toxicity, and excessive light, throughout their growth and development, all of which severely restrict crop growth and yield formation [1,2]. Under drought conditions, plants experience impaired water metabolism, reduced stomatal conductance and photosynthetic efficiency, and excessive accumulation of reactive oxygen species (ROS), resulting in cellular oxidative damage [3]. Salinity stress induces osmotic stress and ionic toxicity caused by the excessive accumulation of Na^+^ and Cl^−^ in soil, thereby disrupting cell membrane integrity, intracellular osmotic homeostasis, biosynthetic processes, and energy metabolism [4]. Low-temperature stress inhibits the activities of key metabolic enzymes, damages chloroplast structures, impairs normal plant growth, and may ultimately cause plant death under severe conditions [5,6]. To mitigate the adverse effects of cold, drought, salinity, and other environmental stresses, plants have evolved sophisticated molecular regulatory networks that coordinate the expression of diverse functional genes and transcription factors involved in stress perception, signal transduction, and defense responses [7]. Therefore, systematic identification and characterization of stress-responsive gene families are important for elucidating the molecular mechanisms underlying plant abiotic stress responses and identifying valuable genetic resources for improving stress tolerance [8].

The LOR gene family is widely distributed across plants, fungi, bacteria, and archaea and is characterized by the presence of a highly conserved LOR domain (Pfam 04525) [9,10,11]. LOR proteins exhibit a conserved structural architecture consisting of a 12-stranded β-barrel surrounding a central C-terminal α-helix, which resembles the C-terminal domain of Tubby proteins. These conserved β-barrel and α-helix structures are involved in protein–protein and protein–DNA interactions and may contribute to the regulation of protein stability, chromatin organization, and downstream gene transcription [12,13,14]. The LOR gene family was named after its first identified member in *Arabidopsis thaliana*, *AtLURP1* (Late Upregulated in Response to *Hyaloperonospora parasitica* 1). The *Arabidopsis* LOR family contains 20 members, among which *AtLURP1* and *AtLOR1* have been the most extensively characterized. As a well-established salicylic acid (SA)-responsive marker gene, *AtLURP1* is strongly induced by pathogen infection, participates in SA-mediated plant immunity, negatively regulates pathogen-induced cell death, and enhances resistance to multiple biotic stresses [15]. *AtLOR1* is constitutively expressed and functions as an important component of basal plant defense [16]. In addition to their roles in biotic stress responses, LOR family members in *Glycine max* and *Brassica napus* have been reported to participate in drought and salt stress responses, as well as in ABA signaling pathways [17,18]. However, the systematic structural characteristics, expression patterns, and biological functions of the LOR gene family under diverse abiotic stresses remain largely unexplored in most plant species.

*Solanum lycopersicoides*, a wild tomato species native to the Andes Mountains along the border between Chile and Peru, is closely related to cultivated tomato. Compared with cultivated tomato, *S. lycopersicoides* exhibits remarkable cold tolerance and disease resistance. It is capable of flowering and fruiting normally at temperatures ranging from −1.25 °C to 5.3 °C, making it a valuable wild germplasm resource for identifying stress-tolerance genes and improving abiotic stress tolerance in cultivated tomato. To date, no systematic study has investigated the LOR gene family in *S. lycopersicoides*, and the biological functions of these genes in abiotic stress responses remain unclear. In this study, genome-wide screening of the *S. lycopersicoides* genome identified 19 *ScLOR* genes. We subsequently performed comprehensive analyses of their phylogenetic relationships, gene structures, conserved motifs, and promoter cis-elements, and investigated their expression patterns under cold, drought, and salt stresses. Furthermore, the functional behaviour of the key candidate gene *ScLOR16* was characterized under cold and drought conditions. This study provides new insights into the stress-responsive functions of the LOR gene family and lays a foundation for future research on *Solanum lycopersicoides* stress-adaptation mechanisms.

## 2. Materials and Methods

### 2.1. Plant Materials and Stress Treatments

The introgression line LA4259 of the wild tomato relative *Solanum lycopersicoides* was provided by the Horticultural Crop Biotechnology Laboratory, Northeast Agricultural University. LA4259 is a *Solanum lycopersicoides* introgression line developed using the cultivated tomato VF36 as the female parent and *S. lycopersicoides* LA2951 as the male parent. Seeds were first soaked in water at 55 °C for 10 min and then placed in an incubator at 30 °C under continuous darkness for germination. Germinated seeds were sown in a substrate composed of perlite, vermiculite, and nutrient soil at a volume ratio of 1:1:1. Seedlings were cultivated in a controlled growth chamber under a 16 h light/8 h dark photoperiod, with day/night temperatures of 25 °C/22 °C. The growth chamber was maintained at a photosynthetic photon flux density (PPFD) of 400 μmol·m^−2^·s^−1^ and a relative air humidity of approximately 65–70%. Each seedling was grown in a container measuring 7 cm × 7 cm × 8 cm. Pure water and half-strength Hoagland’s solution were alternately supplied for irrigation. When the seedlings reached the five-leaf stage (approximately 28 days old), they were transferred to an incubator set at 4 °C for cold-stress treatment under otherwise identical light conditions.

Seedlings at the same developmental stage were hydroponically pre-cultured in quarter-strength Hoagland’s solution for two days. After pre-cultivation, a 200 mM NaCl solution was applied to induce salt stress, while 20% PEG6000 was used to generate osmotic stress as a partial simulation of drought stress. Leaf samples (100 mg) were collected at 0, 1, 3, 6, 12, 24, and 36 h after treatment, immediately frozen in liquid nitrogen, and stored at −80 °C. Three biological replicates were included for each time point, with one independent seedling sampled per biological replicate. Distinct individual plants were used as biological replicates at different stress time points. Twenty-one seedlings were used for each stress treatment across the seven sampling time points, resulting in a total of 63 seedlings for the three stress treatments. Sampling was performed using the penultimate fully expanded true leaf at the five-leaf seedling stage, and the same leaf position was maintained across all sampling time points. Tissue samples, including roots, stems, leaves, flowers, fruits, and seeds, were harvested under the same cultivation conditions and with the same sampling quantities for subsequent tissue-specific expression analysis.

### 2.2. Genome-Wide Identification of LOR Family Members in Solanum lycopersicoides

The genomic sequence and gene annotation files of *S. lycopersicoides* were obtained from the Horticultural Crop Biotechnology Laboratory, Northeast Agricultural University. Previous studies have demonstrated that Pfam04525 corresponds to the conserved domain of the LOR gene family. Therefore, the hidden Markov model (HMM) profile of PF04525 was downloaded from the InterPro database (https://www.ebi.ac.uk/interpro/; accessed on 28 July 2025) [19,20].

The hmmsearch program (v3.4) was employed to identify candidate LOR protein sequences using the GA threshold and an E-value cutoff of <1 × 10^−10^. Candidate sequences were further validated for conserved domains using SMART (https://smart.embl.de/; accessed on 30 July 2025), the NCBI Conserved Domain Database (CDD) (https://www.ncbi.nlm.nih.gov/Structure/cdd/wrpsb.cgi; accessed on 30 July 2025), and the Pfam database (https://www.ebi.ac.uk/interpro/; accessed on 30 July 2025). The resulting non-redundant sequences were considered reliable LOR family members. Subcellular localization was predicted using WoLF PSORT (https://www.genscript.com/wolf-psort.html; accessed on 30 July 2025). Protein molecular weights and theoretical isoelectric point (pI) values were calculated using ExPASy (https://www.expasy.org/; accessed on 30 July 2025).

### 2.3. Analysis of Gene Structures and Conserved Protein Motifs

The exon–intron structures and conserved protein motifs of the *ScLOR* family members were analysed. Briefly, TBtools v2.483 was used to align the coding sequences (CDSs) with the corresponding genomic sequences of the predicted *ScLOR* genes to characterise their exon–intron structures. Conserved motifs were identified using MEME (http://meme-suite.org/; accessed on 10 August 2025) with the following parameters: zero or one occurrence of each motif per sequence, a maximum of 10 motifs, and motif lengths ranging from 6 to 50 amino acids. The conserved motifs and exon–intron structures were integrated and visualised using TBtools v2.483.

### 2.4. Prediction of Cis-Acting Regulatory Elements in Promoter Regions

The 2000-bp genomic sequences upstream of the translation initiation codon (ATG) of each *ScLOR* gene were extracted as promoter regions. The PlantCARE database (http://bioinformatics.psb.ugent.be/webtools/plantcare/html/; accessed on 26 August 2025) was used to identify cis-regulatory elements within these promoter sequences. After screening for major functional elements, TBtools v2.483 was used for data visualisation and figure generation.

### 2.5. Phylogenetic Analysis of LOR Proteins

LOR protein sequences from *Arabidopsis thaliana* and *Brassica napus* were retrieved from published studies [17]. Full-length protein sequences were aligned using MUSCLE v3.8.31, and poorly conserved regions were trimmed using trimAl v1.4.rev22 with the automated1 parameter. A maximum-likelihood phylogenetic tree was constructed using IQ-TREE2 v2.2.2, which integrates ModelFinder for automatic substitution model selection. Branch support values were assessed using 1000 bootstrap replicates. The phylogenetic tree was visualised and refined using Evolview (https://www.evolgenius.info/evolview/; accessed on 30 August 2025).

### 2.6. Chromosomal Localisation and Collinearity Analysis

TBtools v2.483 was used to determine the chromosomal positions of the *ScLOR* genes based on gene ID information. All *ScLOR* genes were renamed according to their chromosomal locations. The GFF files and whole-genome sequences of *Arabidopsis thaliana* and *Solanum lycopersicum* were downloaded from the Ensembl database. The MCScanX module integrated into TBtools v2.483 was used to analyse interspecific collinearity between *S. lycopersicoides* and the two reference species.

### 2.7. Expression Profiling of ScLOR Family Members

Total RNA was extracted from tomato leaves using FreeZol reagent (Vazyme, Nanjing, China), and first-strand cDNA was synthesised using the HiScript III RT SuperMix kit (Vazyme, Nanjing, China) for analysis of the expression patterns of *ScLOR* genes. RT-qPCR primers were designed using NCBI Primer-BLAST (https://www.ncbi.nlm.nih.gov/tools/primer-blast/; accessed on 17 November 2025), and the primer sequences are listed in Table S1.

RT-qPCR was performed using ChamQ Universal SYBR qPCR Master Mix (Vazyme, Nanjing, China) in a 20 μL reaction mixture containing 10 μL of 2× ChamQ Universal SYBR qPCR Master Mix, 0.5 μL of forward primer, 0.5 μL of reverse primer, 1.0 μL of cDNA template, and 8 μL of nuclease-free water. Amplification was performed on a QuantStudio 3 Real-Time PCR System using the following programme: initial denaturation at 95 °C for 30 s, followed by 40 cycles of 95 °C for 5 s and 60 °C for 30 s. Relative expression levels were calculated using the 2^−ΔΔCt^ method [21], with *Scβ-Actin* used as the internal reference gene. Three biological replicates were analysed for each sample. A single sharp melting peak was observed for all amplicons, confirming specific target amplification without nonspecific products or primer dimers.

### 2.8. Subcellular Localisation

The full-length coding sequence of *ScLOR16* was amplified from *S. lycopersicoides* and cloned into the transient expression vector pSuper1300-GFP to investigate the subcellular localisation of ScLOR16. *Agrobacterium tumefaciens* GV3101 harbouring pSuper1300-*ScLOR16*-GFP was co-infiltrated with GV3101 carrying a nuclear marker construct. Empty pSuper1300-GFP co-infiltrated with the nuclear marker strain was used as the control. The mixed bacterial suspensions were infiltrated into tobacco leaves. Fluorescence signals from pSuper1300-*ScLOR16*-GFP and the nuclear marker were observed using a laser-scanning confocal microscope (TCS SP8, Carl Zeiss, Jenna, Germany) to determine the subcellular localisation of ScLOR16.

### 2.9. VIGS of ScLOR16

The coding sequence of *ScLOR16* was submitted to the SGN VIGS Tool (https://vigs.solgenomics.net/; accessed on 15 January 2026) to identify a 300-bp fragment suitable for VIGS-mediated gene silencing (Table S2). The recombinant silencing vector pTRV2-ScLOR16 was constructed and introduced into competent *A. tumefaciens* GV3101 cells, together with empty pTRV1, empty pTRV2, and the positive-control vector pTRV2-*PDS*. *Agrobacterium* cultures containing pTRV1 were mixed at a 1:1 volume ratio with cultures harbouring pTRV2, pTRV2-*ScLOR16*, or pTRV2-*PDS* and incubated in darkness at 28 °C for 3 h. The mixed bacterial suspensions were infiltrated into the abaxial surfaces of cotyledons from one-week-old tomato seedlings. Following infiltration, the plants were maintained in darkness at 20 °C for 24 h and subsequently transferred to greenhouse conditions (25 °C/22 °C day/night temperature, 16 h light/8 h dark photoperiod) for continued growth. Seedlings infiltrated with empty pTRV2 were used as negative controls. When pTRV2-*PDS*-infiltrated indicator plants exhibited leaf-albino phenotypes, RT-qPCR was performed to identify plants with high silencing efficiency for subsequent stress treatments. A total of 20 plants were subjected to VIGS treatment, of which 14 met the >50% silencing-efficiency criterion, and 12 independent plants were ultimately included in the physiological analyses.

### 2.10. Determination of Antioxidant Enzyme Activities and Physiological Indicators

Plants with silenced *ScLOR16* (pTRV2-*ScLOR16*) and empty-vector control plants were subjected to cold stress at 4 °C for 24 h. For drought treatment, both the empty-vector control and *ScLOR16*-silenced groups were fully watered to soil saturation on day 0, followed by continuous water withholding for 4 days under identical growth conditions. Leaf samples (100 mg fresh weight) were collected upon completion of each stress treatment. Three biological replicates (individual plants) were collected for the empty-vector control and *ScLOR16*-silenced groups at each sampling time point. In total, 12 plants were used for the VIGS-based functional assays, covering the two stress treatments and two sampling time points. Superoxide dismutase (SOD) activity, peroxidase (POD) activity, thiobarbituric acid-reactive substances (TBARS) content, and proline (Pro) content were measured using commercial assay kits (Grace Biotechnology, Suzhou, China), strictly according to the manufacturer’s instructions. All physiological parameters were calculated based on fresh weight.

### 2.11. Statistical Analysis

All experiments were performed with three independent biological replicates unless otherwise stated. Data are presented as the mean ± standard error (SE). Statistical analyses of the relative expression levels of *ScLOR* genes under cold, drought, and salt stress across different time points were performed using GraphPad Prism 9.0. One-way ANOVA was applied to evaluate the data, followed by Dunnett’s multiple-comparison test, in which each stress-treatment time point was compared with the 0 h control. Differences with *p* < 0.05 were considered statistically significant.

The effects of *ScLOR16* silencing status, cold or drought stress duration, and their interaction on physiological indices were analysed using GraphPad Prism 9.0. Ordinary two-way ANOVA was used for the analysis, followed by Tukey’s test for pairwise comparisons. Differences with *p* < 0.05 were considered statistically significant.

## 3. Results

### 3.1. Identification of ScLOR Genes in Solanum lycopersicoides

A total of 19 *ScLOR* genes were identified in the *S. lycopersicoides* genome. All deduced protein sequences were submitted to the SMART, NCBI Conserved Domain Database (CDD), and Pfam databases to confirm the presence of the conserved LOR domain. The identified genes were designated *ScLOR1* to *ScLOR19* according to their chromosomal positions.

The physicochemical properties and predicted subcellular localisations of the ScLOR proteins are summarised in Table 1. The protein lengths ranged from 173 amino acids (ScLOR8) to 266 amino acids (ScLOR15), with molecular weights ranging from 19.62 to 30.22 kDa and theoretical isoelectric point (pI) values ranging from 5.18 (ScLOR1) to 9.94 (ScLOR15). Among the 19 ScLOR proteins, only three were predicted to be acidic, whereas the remaining proteins were predicted to be basic. The instability index values of all ScLOR proteins were below 52.

Subcellular localisation analysis indicated that most ScLOR proteins were predicted to localise predominantly to chloroplasts, with additional predicted localisation in the nucleus, cytoplasm, mitochondria, and other cellular compartments.

### 3.2. Phylogenetic Relationships of LOR Proteins from Solanum lycopersicoides, Arabidopsis thaliana and Brassica napus

To investigate the evolutionary relationships of the LOR superfamily, a maximum-likelihood phylogenetic tree was constructed based on LOR protein sequences from *S. lycopersicoides*, *Arabidopsis thaliana*, and *Brassica napus*. The phylogenetic analysis included 19 identified ScLOR proteins from *S. lycopersicoides*, 20 AtLOR proteins from the model plant *A. thaliana*, and 56 BnLOR proteins from *B. napus* (Table S3).

**Figure 1:**
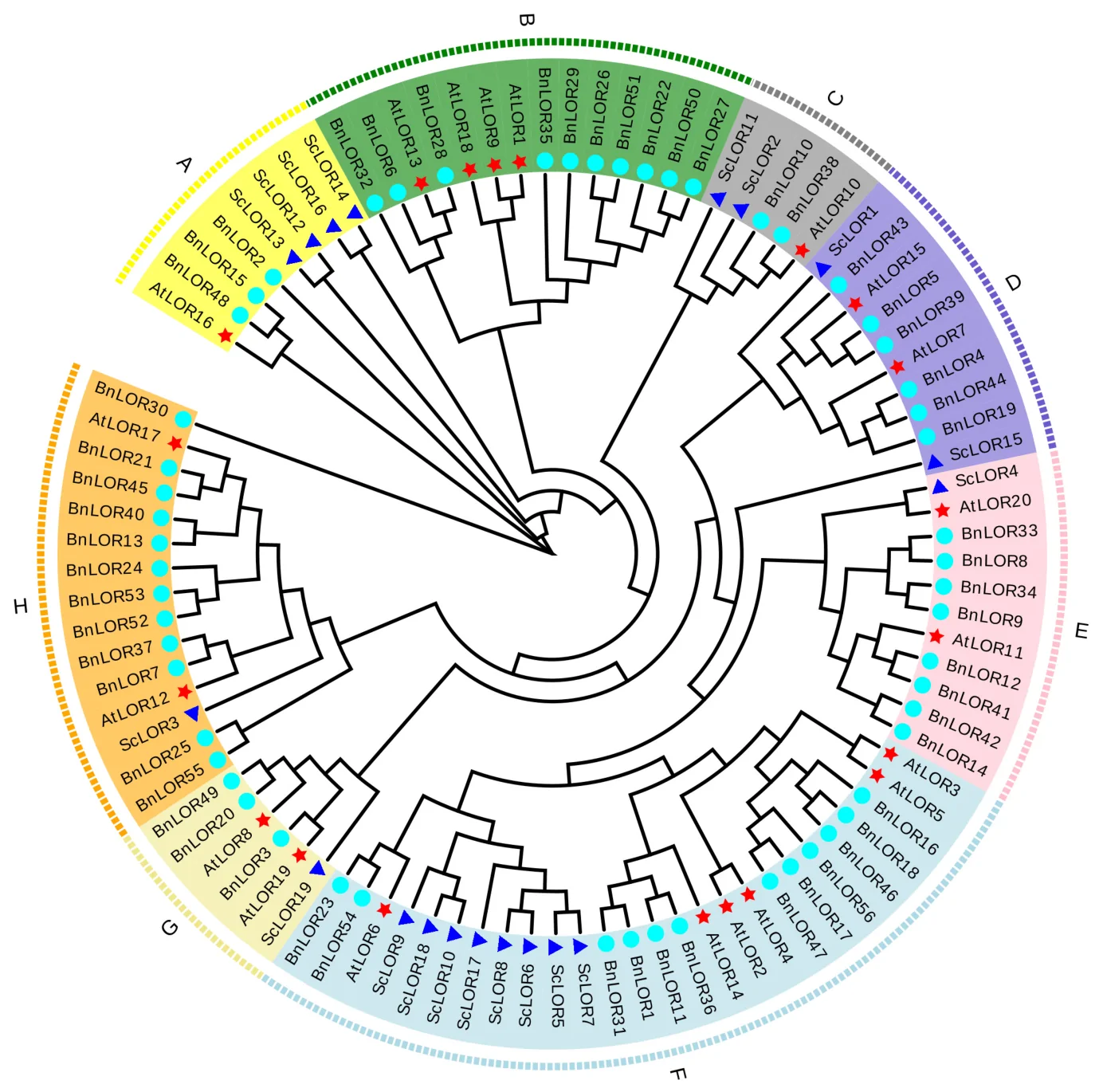
Phylogenetic analysis of LOR proteins from *Solanum lycopersicoides*, *Arabidopsis thaliana* and *Brassica napus* was performed. A maximum-likelihood phylogenetic tree was constructed, and branch support values were assessed with 1000 bootstrap replicates. Blue triangles represent members of the *ScLOR* family, red pentagrams denote *AtLOR* family, and cyan circles indicate *BnLOR* family members. Based on phylogenetic clustering, the tree was divided into eight subgroups (A–H), each distinguished by a distinct background colour.

The phylogenetic tree showed that LOR proteins from the same species tended to cluster together (Figure 1). Based on the tree topology, the LOR proteins were classified into eight subgroups (A–H). Subgroups B, F, and H contained the largest numbers of members, with 14, 26, and 15 LOR proteins, respectively, whereas subgroups A, C, D, E, and G contained 5–11 members each.

Overall, LOR proteins within the same subgroup exhibited similar evolutionary relationships, which may provide valuable clues for predicting the potential biological functions of LOR family members in *S. lycopersicoides*.

### 3.3. Gene Structures and Conserved Motifs of the ScLOR Family

**Figure 2:**
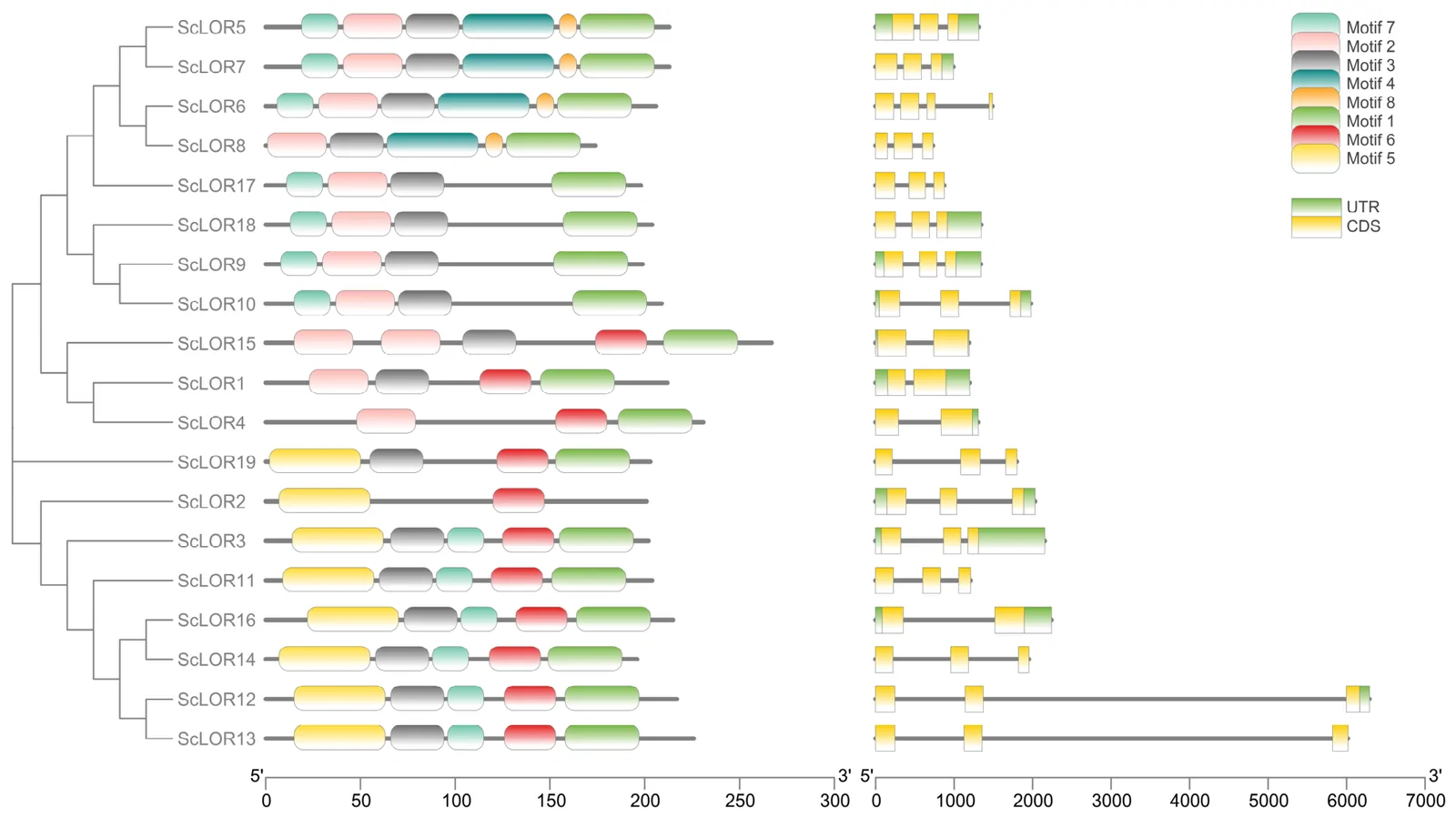
Conserved protein motifs and gene structures of the LOR family in *S. lycopersicoides*. Exon–intron structures of *ScLOR* genes: the relative position and length of exons can be estimated using the scale bar at the bottom. Yellow boxes, grey lines and green boxes represent exons, introns and UTRs, respectively. Putative conserved motifs within ScLOR proteins: distinct motifs and their relative positions are displayed as coloured boxes.

The gene structures and conserved protein motifs of the *ScLOR* family were comprehensively analysed based on the genomic annotation information and protein sequences of *S. lycopersicoides*. The number of introns in the *ScLOR* genes ranged from 1 to 3, whereas the number of exons ranged from 2 to 4. Most *ScLOR* genes contained three exons (Figure 2).

Furthermore, the 19 ScLOR protein sequences were analysed using MEME to identify conserved motifs, resulting in the identification of eight conserved motifs (Figure 2). Among these motifs, Motifs 1 and 3 were highly conserved and represented core components of the *ScLOR* family. Motif 1 was located at the C-terminus of the ScLOR proteins, whereas Motif 3 was located in the central region. Motifs 4 and 8 were predominantly enriched in subgroup F. Proteins belonging to subgroups A, C, and H exhibited highly similar motif compositions, with the exception of ScLOR2.

These results suggest that structural variations among LOR subgroups in *S. lycopersicoides* may contribute to functional diversification among family members.

### 3.4. Identification of Cis-Acting Regulatory Elements in ScLOR Promoters

**Figure 3:**
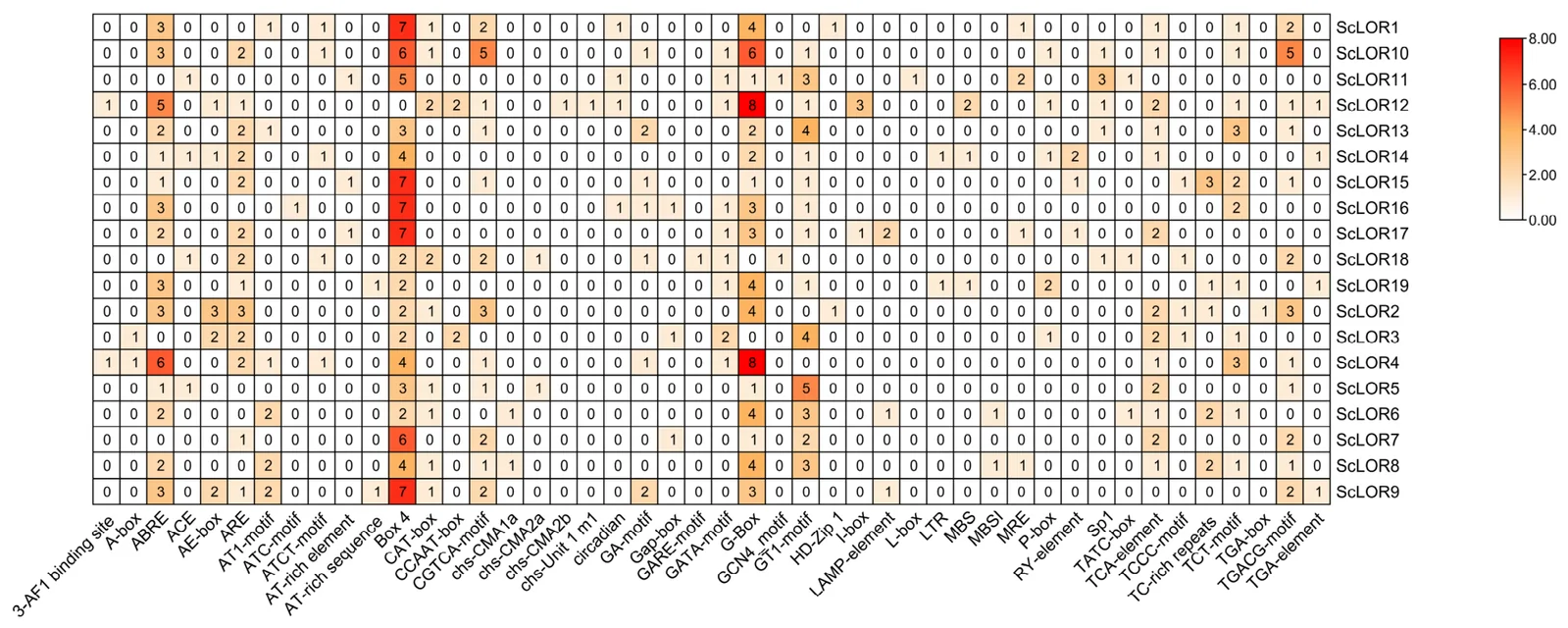
Distribution of cis-acting elements in the promoters of *ScLOR* genes. The 2000 bp upstream sequences of each *ScLOR* gene were analysed. Different types of cis-acting elements are listed at the bottom, and the colour of each rectangle together with the labeled number indicates the quantity of the corresponding cis-element.

A total of 46 types of cis-regulatory elements were identified within the 2000-bp upstream promoter regions of the 19 *ScLOR* genes (Table S4). Their distribution patterns and abundances were visualised using heatmaps (Figure 3). These cis-regulatory elements were classified into four functional categories: core promoter elements, hormone-responsive elements, stress-responsive elements, and light-responsive elements.

Hormone-responsive cis-elements were abundant and widely distributed across the *ScLOR* promoter regions. The ABA-responsive element ABRE was identified in 15 of the 19 promoters, with up to six copies detected in a single promoter, suggesting a potential association between the *ScLOR* family and ABA-mediated regulatory pathways. Other hormone-related elements included the SA-responsive TCA element; the MeJA-responsive CGTCA and TGACG motifs; the GA-responsive GARE and P-box elements; and the auxin-responsive TGA elements, all of which were detected in most *ScLOR* promoters.

Regarding stress-responsive elements, the cold-responsive LTR, drought-responsive MBS, anaerobic-responsive ARE, and defence-related TC-rich repeats were frequently identified in *ScLOR* promoters, suggesting that *ScLOR* genes may be involved in responses to diverse environmental stresses. In addition, light-responsive motifs, including Box 4, G-box, GT1-motif, and I-box, were widely distributed, with multiple copies detected in almost all *ScLOR* promoters.

### 3.5. Chromosomal Localisation and Synteny Analysis of ScLOR Genes

**Figure 4:**
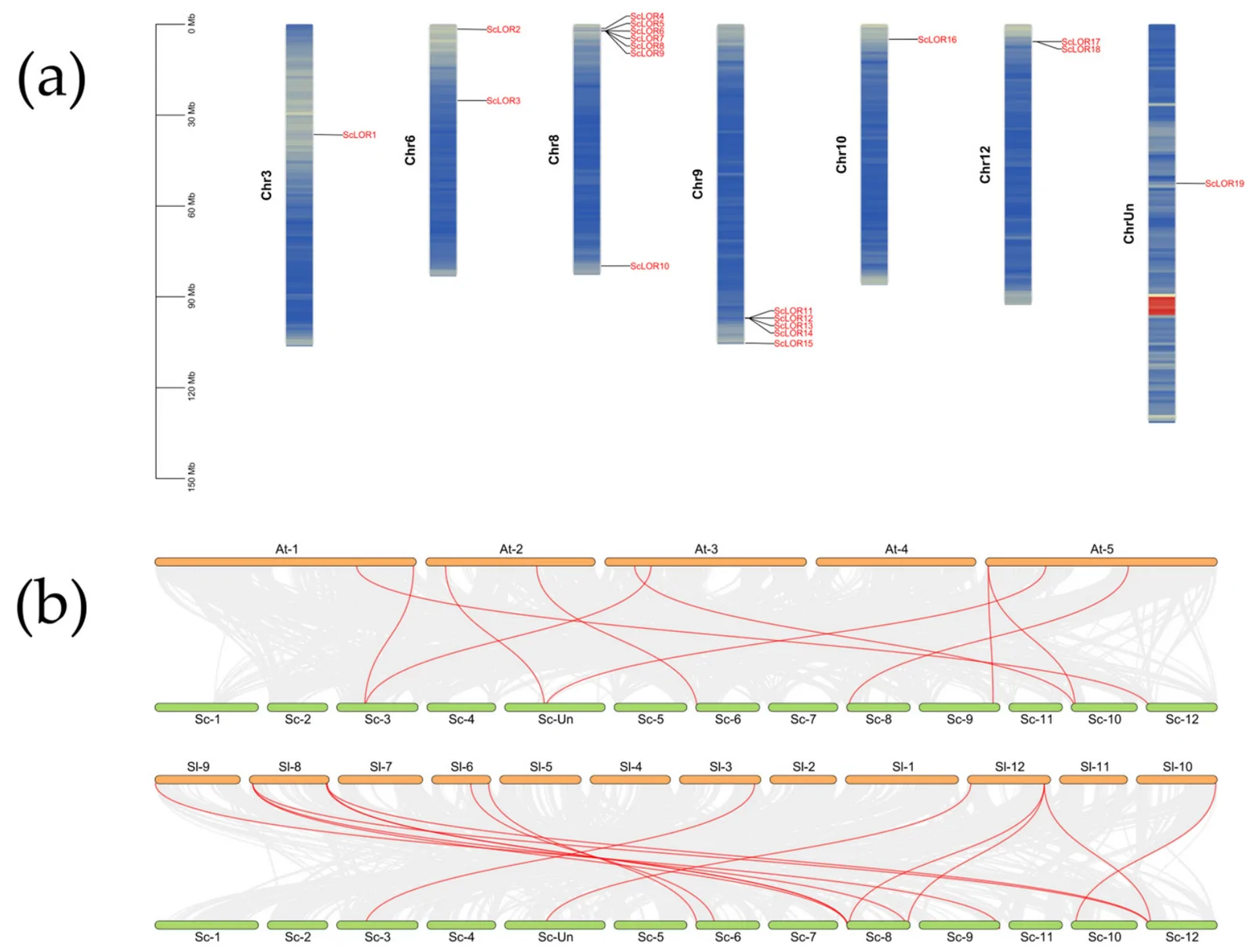
Genome-wide chromosomal mapping and synteny analysis of the *ScLOR* gene family. (**a**) Chromosomal distribution of all *ScLOR* genes in the *Solanum lycopersicoides* genome. The scale bar on the left is marked in megabase (Mb) units. (**b**) Interspecies collinear relationships of LOR genes among *Solanum lycopersicoides*, *Solanum lycopersicum* and *Arabidopsis thaliana*. Collinear gene pairs are connected by red lines.

TBtools v2.483 was used to analyse the chromosomal distribution of *ScLOR* genes in *S. lycopersicoides*. The genes were renamed *ScLOR1* to *ScLOR19* according to their chromosomal coordinates for ease of identification. The 19 *ScLOR* genes were unevenly distributed across the chromosomes of *S. lycopersicoides* (Figure 4a). Most *ScLOR* genes were located on chromosomes 8 and 9, whereas chromosomes 1, 2, 4, 5, 7, and 11 contained no *ScLOR* genes.

MCScanX was employed to perform synteny analysis among *S. lycopersicoides*, *Solanum lycopersicum*, and *Arabidopsis thaliana* to investigate the evolutionary relationships of LOR family members. A total of 25 syntenic gene pairs were identified, including 10 collinear pairs between *S. lycopersicoides* and *A. thaliana* and 15 collinear pairs between *S. lycopersicoides* and *S. lycopersicum* (Figure 4b). Chromosome 8 contained the largest number of syntenic gene pairs.

### 3.6. Expression Profiles of ScLOR Genes Under Cold, Drought and Salt Stresses in the Solanum lycopersicoides Introgression Line LA4259

Cold, drought and salt stress treatments were applied to five-leaf-stage seedlings of the *Solanum lycopersicoides* introgression line LA4259. Cold treatment was performed in a 4 °C incubator under the same light conditions, whereas drought and salt treatments were applied through *hydroponic* culture supplemented with 20% PEG6000 and 200 mM NaCl, respectively, to simulate drought and salinity stresses. RT-qPCR was performed to analyse the temporal expression patterns of all *ScLOR* genes at seven time points (0, 1, 3, 6, 12, 24, and 36 h) after stress treatment.

**Figure 5:**
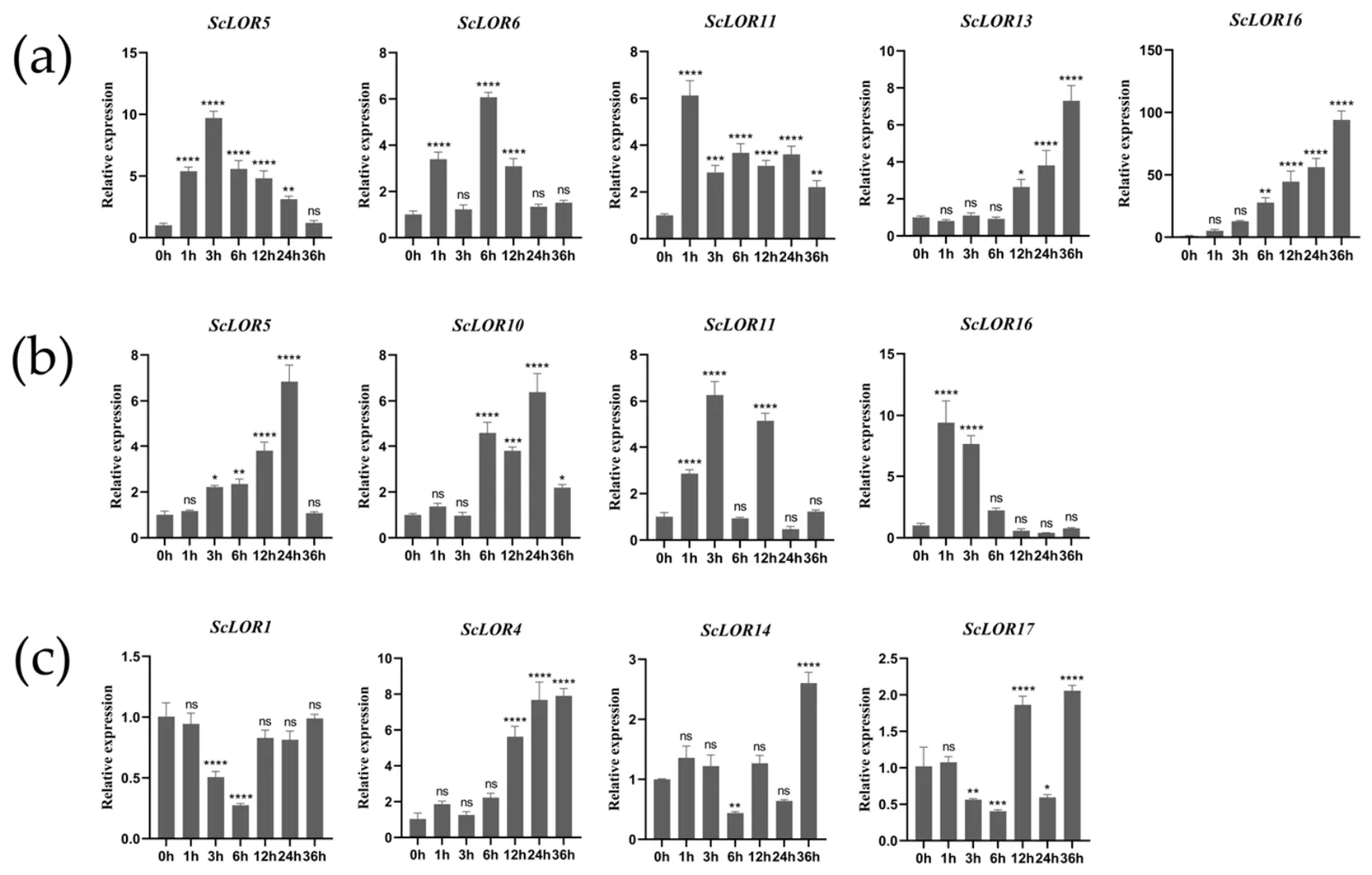
Expression patterns of *ScLOR* genes under cold, drought and salt stresses determined by RT-qPCR. (**a**) Genes with significantly altered expression of *ScLOR* under cold treatment; subscripts indicate cold treatment durations. (**b**) Genes with significantly altered expression of *ScLOR* under drought stress simulated by 20% PEG6000; subscripts represent treatment durations with 20% PEG6000. (**c**) Genes with significantly altered expression of *ScLOR* under high-salt stress with 200 mM NaCl; subscripts denote treatment durations with 200 mM NaCl. Three biological replicates were performed. All expression values were normalized to the 0 h control group. Statistical significance was marked with ns (non-significant), *, **, ***, and ****, corresponding to *p* < 0.05, *p* < 0.01, *p* < 0.001, and *p* < 0.0001, respectively. ns represents no significant difference (*p* ≥ 0.05). Data are presented as mean ± standard error of the mean (SEM, *n* = 3).

Distinct stress-responsive expression patterns were observed among different *ScLOR* members under cold, drought, and salt treatments. Under cold stress, the transcript levels of *ScLOR5*, *ScLOR6*, *ScLOR11*, *ScLOR13*, and *ScLOR16* were significantly upregulated with prolonged treatment, exhibiting typical cold-induced expression patterns. Among these genes, *ScLOR16* showed the strongest induction, suggesting that it may represent a key cold-responsive candidate gene (Figure 5a).

Following drought treatment, *ScLOR5*, *ScLOR10*, *ScLOR11*, and *ScLOR16* were significantly induced and exhibited drought-responsive expression patterns (Figure 5b). Under salt stress, *ScLOR1* expression was markedly downregulated, whereas *ScLOR4* was strongly induced. In addition, *ScLOR14* and *ScLOR17* displayed dynamic fluctuation patterns during salt treatment (Figure 5c).

Overall, different *ScLOR* members exhibited distinct expression patterns under abiotic stresses, suggesting functional diversification within the *ScLOR* family. Notably, *ScLOR16* was strongly induced by both cold and drought stresses, providing expression evidence supporting its subsequent functional validation using VIGS (Table S5).

### 3.7. Tissue-Specific Expression and Subcellular Localisation of ScLOR16

**Figure 6:**
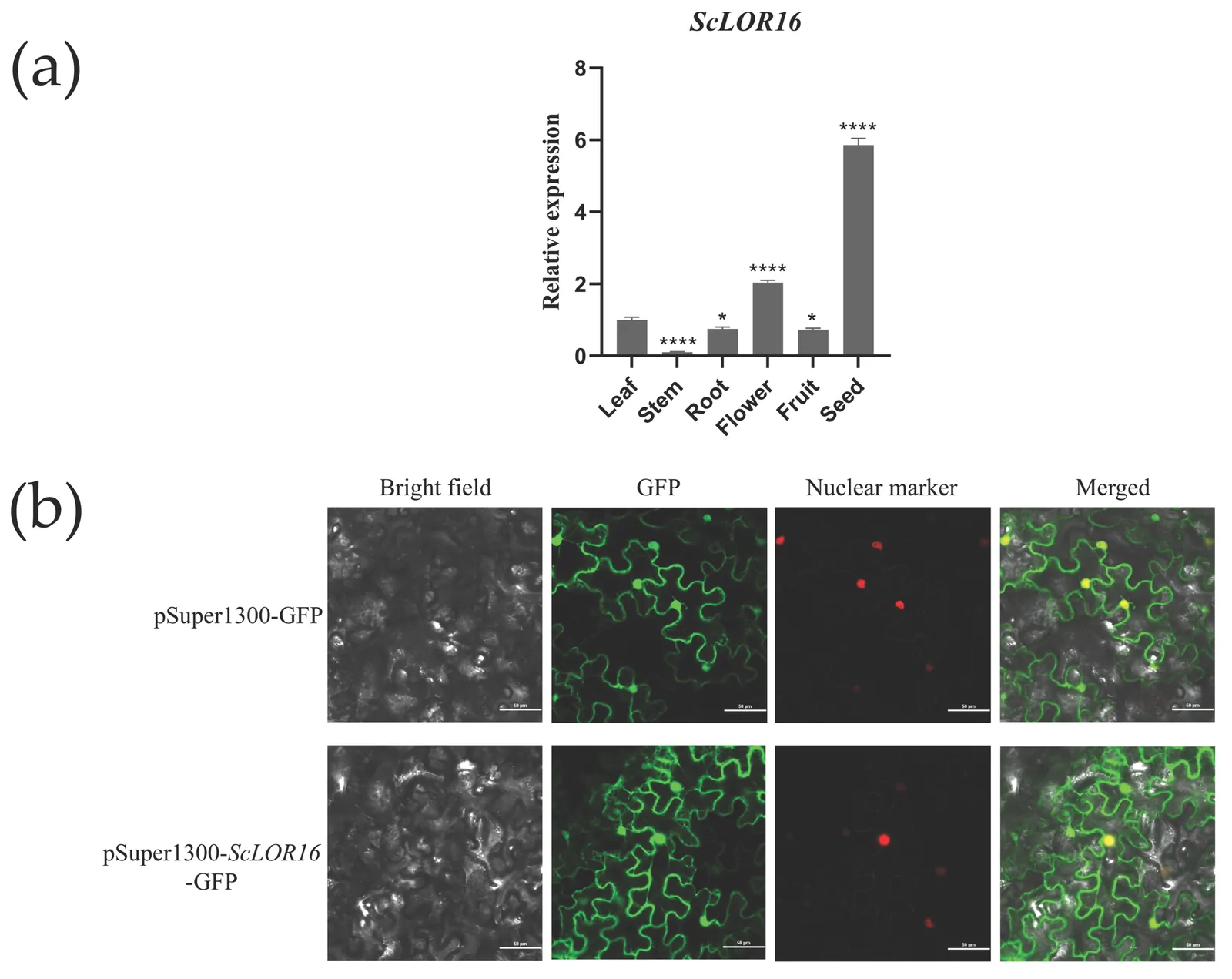
Tissue expression pattern and subcellular localization of *ScLOR16*. (**a**) Relative expression levels of *ScLOR16* in various tissues (roots, stems, leaves, flowers, fruits, and seeds) of *Solanum lycopersicoides*. Gene expression values were normalized using leaf samples as the control. Data are presented as mean ± SEM (*n* = 3). Significance is indicated by asterisks: *, *p* < 0.05; ****, *p* < 0.0001. (**b**) Subcellular localization of the ScLOR16 protein in tobacco epidermal cells. The recombinant construct pSuper1300-*ScLOR16*-GFP was transiently expressed in tobacco leaves. Confocal microscopy demonstrated that the ScLOR16 fusion protein accumulates in both the nucleus and cytoplasm. GFP, green fluorescence channel; Nuclear marker, nuclear localization marker fluorescence channel; bright field, transmitted light channel; merged, overlay image of GFP, nuclear marker and bright field signals.

Based on RT-qPCR analysis under cold, drought, and salt stress conditions, *ScLOR16* was selected as a candidate gene for subsequent functional validation. RT-qPCR was further performed to investigate the tissue-specific expression pattern of *ScLOR16* in the roots, stems, leaves, flowers, fruits, and seeds of *S. lycopersicoides*. The results showed that *ScLOR16* exhibited the highest transcript abundance in seeds, followed by flowers and leaves, whereas relatively low expression levels were detected in stems (Figure 6a, Table S6).

To determine the subcellular localisation of ScLOR16, the full-length coding sequence of *ScLOR16* was fused to the pSuper1300-GFP vector and transiently expressed in tobacco leaves (Figure 6b). Confocal microscopy revealed that the *ScLOR16*-GFP fusion protein was localised in both the nucleus and cytoplasm.

### 3.8. Functional Characterisation of ScLOR16 Under Cold and Drought Stress

**Figure 7:**
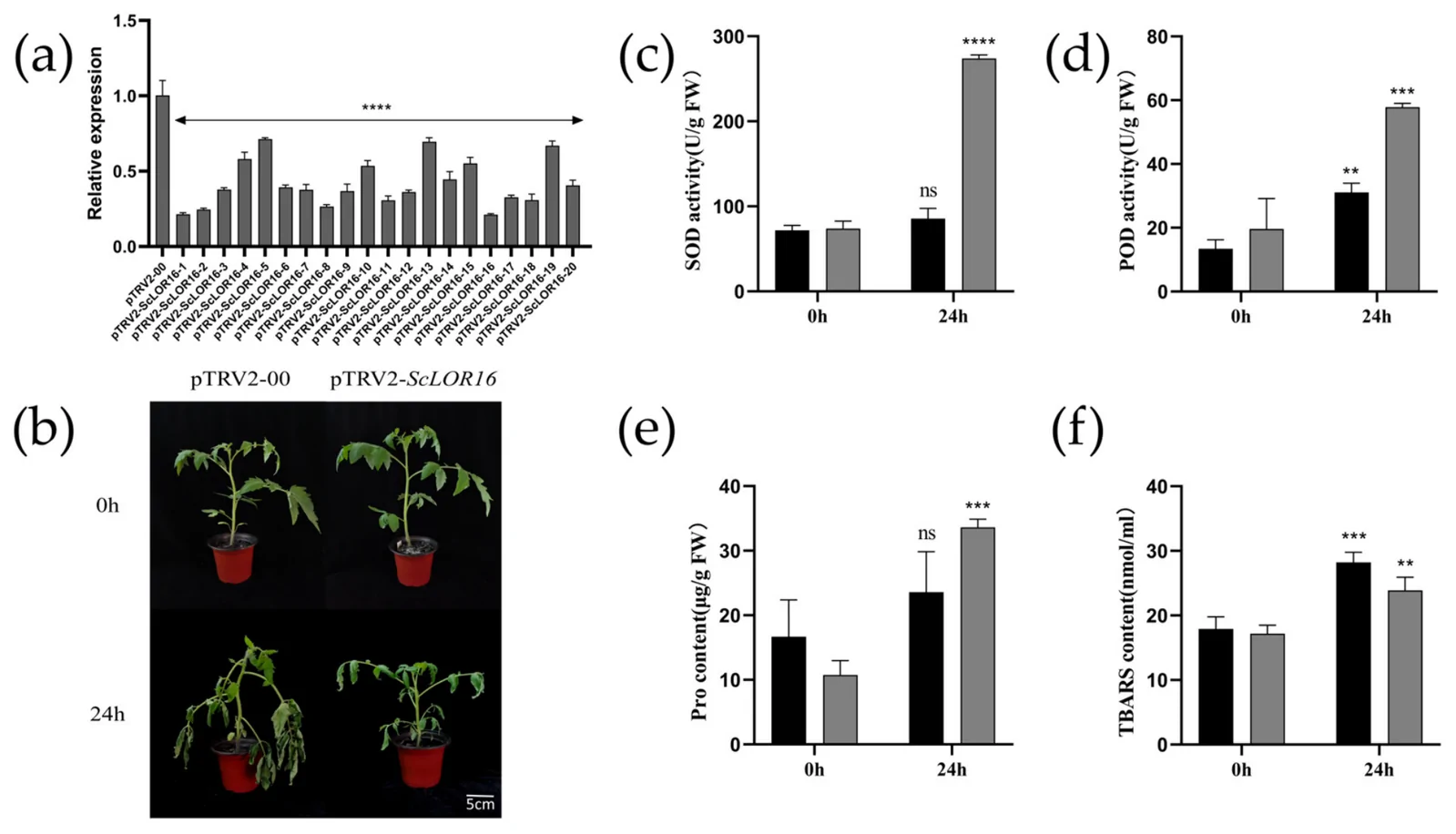
Functional analysis of *ScLOR16* under cold stress via VIGS-mediated gene silencing. (**a**) Detection of *ScLOR16* gene-silencing efficiency. A total of 20 plants from the *ScLOR16*-silenced group (pTRV2-*ScLOR16*) were assayed. Plants with silencing efficiency > 50% were regarded as qualified. The empty-vector control (pTRV2-00) served as the control group. (**b**) Phenotypic differences between empty vector control (pTRV2-00) and *ScLOR16*-silenced (pTRV2-*ScLOR16*) *Solanum lycopersicoides* seedlings after 24 h of 4 °C cold treatment. (**c**–**f**) Physiological index measurements of control and silenced plants before (0 h) and after 24 h cold stress. (**c**) SOD activity; (**d**) POD activity; (**e**) Proline (Pro) content; (**f**) TBARS content. All data are displayed as mean ± SEM (*n* = 3). Statistical differences are labelled with ns (non-significant, *p* ≥ 0.05), **, ***, and ****, corresponding to *p* < 0.01, *p* < 0.001, and *p* < 0.0001, respectively.

RT-qPCR analysis confirmed that *ScLOR16* expression was significantly induced by both cold and drought stresses. To investigate the biological function of *ScLOR16* in abiotic stress responses, VIGS-mediated gene silencing was performed in *Solanum lycopersicoides* introgression line LA4259. When indicator plants exhibited leaf albino phenotypes, the transcript levels of *ScLOR16* were quantified to identify silenced lines showing more than 50% reduction in expression compared with empty vector controls (Figure 7a, Table S7). These silenced lines were subsequently used for stress tolerance assays.

After 24 h of cold treatment at 4 °C, empty vector control plants (pTRV2-00) exhibited more severe wilting symptoms than *ScLOR16*-silenced plants (pTRV2-*ScLOR16*) (Figure 7b). Leaf samples from untreated (0 h) and cold-treated (24 h) empty vector control and silenced plants were collected to determine TBARS and proline contents, as well as SOD and POD activities (Figure 7c–f).

Compared with untreated samples (0 h), SOD and POD activities, as well as proline and TBARS contents, were significantly increased in both groups after 24 h of cold treatment. At the same time point, SOD and POD activities were significantly higher in pTRV2-*ScLOR16*-silenced plants than in empty vector controls. Proline, an important osmoprotective compound involved in cold stress responses, accumulated at significantly higher levels in silenced plants after cold induction. In contrast, TBARS, a widely used indicator of membrane lipid peroxidation, was significantly lower in *ScLOR16*-silenced plants than in control plants after 24 h of cold exposure.

**Figure 8:**
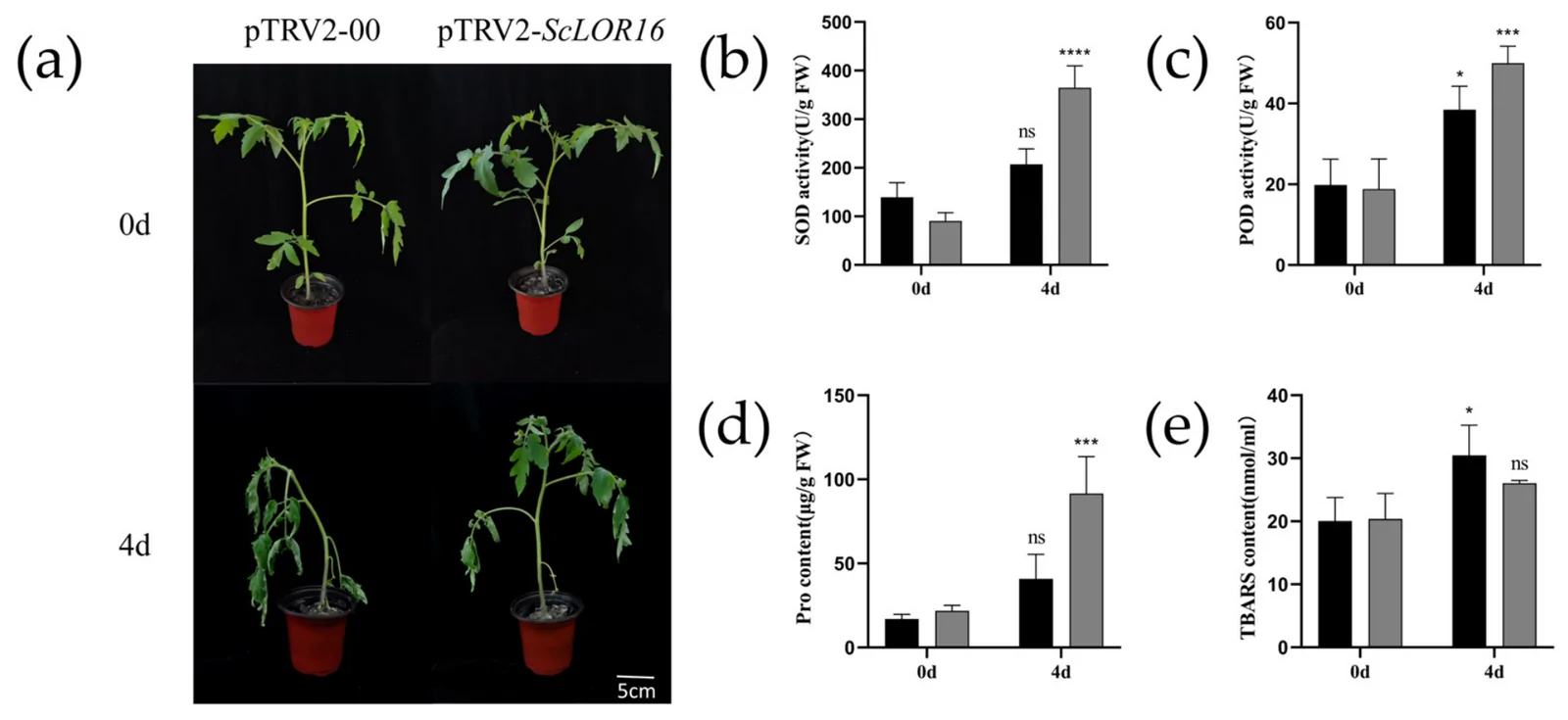
Phenotypic and physiological analysis of empty vector control and *ScLOR16*-silenced *Solanum lycopersicoides* introgression line LA4259 under drought stress. (**a**) Morphological phenotypes of control (pTRV2-00) and *ScLOR16*-VIGS silenced plants after 4 days of drought treatment. (**b**–**e**) Determination of physiological indices in control and silenced leaves collected at 0 d (normal growth condition) and 4 d after drought stress. (**b**) SOD activity; (**c**) POD activity; (**d**) Proline (Pro) content; (**e**) TBARS content. All data are presented as mean ± SEM (*n* = 3). Statistical differences are labelled with ns (non-significant, *p* ≥ 0.05), *, ***, and ****, corresponding to *p* < 0.05, *p* < 0.001, and *p* < 0.0001, respectively.

A parallel drought treatment lasting 4 days was applied to both groups. At the end of the treatment, leaves of empty vector control plants exhibited severe water loss, leaf curling, wilting, and chlorosis, whereas *ScLOR16*-silenced plants displayed milder stress symptoms (Figure 8a). Leaf samples collected at 0 and 4 days after drought treatment were used for physiological indicator measurements (Figure 8b–e).

Compared with the untreated plants (0 d), SOD and POD activities, as well as proline and TBARS contents, were significantly increased in both empty vector control and *ScLOR16*-silenced plants after 4 days of drought stress. Under drought conditions, pTRV2-*ScLOR16* plants exhibited significantly higher SOD and POD activities than empty vector controls. In addition, silenced plants accumulated higher levels of proline, which may contribute to osmotic adjustment under drought stress, whereas TBARS contents were significantly lower, suggesting reduced membrane lipid peroxidation and oxidative damage.

Combined phenotypic and physiological analyses under cold and drought conditions demonstrated that *ScLOR16* negatively regulates cold and drought tolerance in *Solanum lycopersicoides* introgression line LA4259. Silencing of *ScLOR16* significantly enhanced stress-induced antioxidant enzyme activities, promoted proline accumulation, reduced membrane oxidative damage, and alleviated stress-associated wilting symptoms under both cold and drought conditions.

## 4. Discussion

With the rapid accumulation of plant reference genome resources, numerous gene families have been identified and characterised. However, research on the LOR gene family remains relatively limited [22]. In this study, we systematically characterised the LOR gene family in the stress-tolerant wild tomato germplasm *Solanum lycopersicoides*. A total of 19 *ScLOR* family members were identified, and comprehensive bioinformatic analyses were performed, including analyses of physicochemical properties, evolutionary relationships, gene structures, conserved motifs, chromosomal distribution, collinearity, promoter cis-elements, tissue-specific expression patterns, and responses to abiotic stresses. Furthermore, we identified *ScLOR16* as a key cold- and drought-responsive gene and investigated its potential role as a negative regulator of cold and drought tolerance through tissue expression profiling, subcellular localisation analysis, and VIGS-mediated gene silencing. Previous studies on LOR family genes have mainly focused on individual abiotic stresses. In contrast, this study systematically analysed the differential responses of LOR family members to cold, drought, and salt stresses in wild Solanaceae germplasm and provides functional evidence suggesting a potential negative-regulatory effect of the LOR family member *ScLOR16* on abiotic stress tolerance [17,18].

Phylogenetic analysis classified LOR proteins from *Arabidopsis thaliana*, *Brassica napus*, and *S. lycopersicoides* into eight evolutionary subgroups. Members within the same subgroup exhibited highly similar gene structures and conserved motif compositions, consistent with the evolutionary characteristics of LOR families reported in *Glycine max* and foxtail millet (*Setaria italica*). These findings suggest that conserved subgroup differentiation of the LOR family may have occurred before the divergence of dicotyledonous plants [23]. The stress-responsive candidate gene *ScLOR16* was clustered within a distinct monophyletic clade, and most members of this clade contained abundant cold-, drought-, and ABA-responsive cis-elements in their promoter regions, suggesting potential functional conservation and diversification of the LOR family during evolution [24,25].

Regarding gene expansion patterns, the 19 *ScLOR* genes exhibited an uneven distribution across six chromosomes and unanchored scaffolds, with tandem gene clusters identified on chromosome 8. Combined with the collinear relationships observed among *S. lycopersicoides*, *Solanum lycopersicum*, and *A. thaliana*, these results suggest that segmental duplication contributed substantially to the expansion of the *ScLOR* family, whereas tandem duplication may have played a supplementary role. This expansion pattern is consistent with previous reports of LOR families in *B. napus* and *G. max*, indicating that chromosomal duplication events may represent an important evolutionary mechanism contributing to LOR gene copy-number expansion and functional diversification in dicotyledonous plants [26,27].

Analysis of gene structures and conserved motifs revealed that all *ScLOR* genes contain only 1–3 introns, with compact gene architectures characterised by relatively few introns. Previous studies have shown that genes with fewer introns can undergo rapid transcriptional activation, enabling plants to respond rapidly to external stress signals [28,29]. This may help explain the significant upregulation of multiple *ScLOR* genes within a short period following cold and drought treatments in our study. MEME analysis identified eight conserved motifs, among which Motifs 1 and 3 represented core conserved regions shared by all members, corresponding to the canonical LOR structural fold comprising a 12-stranded β-barrel and a C-terminal α-helix. This conserved domain is essential for maintaining the structural conformation of LOR proteins and mediating protein–protein and protein–DNA interactions. Distinct motif profiles among different subgroups further suggest that motif variation may contribute to functional divergence during evolution, consistent with findings on conserved LOR motifs in foxtail millet and *Brassica napus* [30,31].

Promoter cis-element analysis provides an important preliminary approach for predicting potential regulatory mechanisms of stress-responsive genes [32]. In this study, 46 categories of cis-elements were identified within the 2000 bp upstream promoter regions of the 19 *ScLOR* genes. The ABA-responsive element ABRE was widely distributed among most *ScLOR* promoters, accompanied by abundant cold-responsive LTR, drought-responsive MBS, and anaerobic-responsive ARE motifs. These findings suggest that the *ScLOR* family may participate extensively in ABA-associated cold and drought stress signalling pathways [33,34,35], consistent with the promoter characteristics reported for LOR genes in soybean and rapeseed. Notably, the promoter region of the candidate gene *ScLOR16* contained multiple ABRE elements, providing cis-regulatory evidence supporting its potential involvement in both cold and drought responses. Intriguingly, typical cold-responsive LTR and drought-responsive MBS motifs were absent in the *ScLOR16* promoter, indicating that its cold-and drought-inducible expression is not driven by these canonical stress-responsive elements. Its stress responsiveness is most likely mediated through ABRE-dependent ABA signalling, while contributions from other uncharacterized cis-motifs or indirect trans-regulatory cascades cannot be excluded. Furthermore, variations in the composition and abundance of cis-elements among different family members may contribute to the differential stress-responsive expression patterns of individual *ScLOR* genes.

It is generally considered that seeds are relatively less sensitive to cold and drought stresses compared with vegetative tissues. Nevertheless, *ScLOR16* displays its highest expression level in seeds. This expression pattern suggests that, besides its negative regulatory function in cold and drought responses in vegetative tissues, *ScLOR16* may undertake additional seed-specific biological roles. Potential functions may include seed development, maturation, or seed desiccation-related adaptation, which have not been explored in this work. Further seed-related phenotypic characterization and tissue-specific functional studies are needed to dissect its possible roles in seeds.

Bioinformatic prediction in Table 1 suggested that ScLOR16 is primarily localized to chloroplasts and vacuoles, whereas transient GFP-fusion assays revealed its localization in the nucleus and cytoplasm. Such discrepancies between in-silico prediction and experimental observation are not uncommon. Prediction algorithms largely depend on recognizable targeting signal sequences, and ambiguous or incomplete signal motifs in ScLOR16 may give rise to inaccurate computational outputs. In addition, heterologous transient expression in *Nicotiana benthamiana* could potentially introduce minor localization artifacts. Although our GFP-fusion data reflect the subcellular pattern of ScLOR16 under the heterologous transient-expression system, definitive localization in the native LA4259 background awaits further validation using stable transgenic materials.

RT-qPCR profiling under abiotic stress conditions revealed that *ScLOR16* expression was strongly induced by 4 °C cold and 20% PEG6000-induced osmotic stress (mimicking drought conditions), whereas no obvious upregulation was observed under salt stress. These results indicate that *ScLOR16* is a potential candidate gene involved in cold and drought responses. To investigate its in vivo function, VIGS-mediated silencing was performed to suppress *ScLOR16* expression. Phenotypic observations and physiological measurements after 24 h of cold treatment and 4 days of drought treatment showed that *ScLOR16*-silenced plants exhibited substantially milder wilting symptoms than empty vector controls. Compared with untreated plants (0 h), SOD and POD activities, as well as proline and TBARS contents, were significantly increased in both groups following cold and drought exposure. However, *ScLOR16*-silenced plants exhibited significantly higher antioxidant enzyme activities and greater proline accumulation, accompanied by markedly lower TBARS levels compared with control plants. Proline functions as an important compatible osmolyte involved in osmotic adjustment under stress conditions [36,37], while SOD and POD enzymes contribute to ROS scavenging under adverse environmental conditions [38]. TBARS is widely used as an indicator of membrane lipid peroxidation and oxidative damage [39]. It should be noted that all physiological parameters were calculated based on fresh weight. Water loss under drought-related stress may alter plant fresh weight and introduce potential bias to these measurements. Dry-weight-normalized quantification will be adopted in our future experiments to avoid such interference. Collectively, these physiological results indicate that reduced *ScLOR16* expression enhances antioxidant capacity, promotes osmotic adjustment, and alleviates membrane lipid peroxidation under cold and drought stress, indicating that *ScLOR16* may be involved in negatively modulating plant responses to cold and drought stress.

It should be noted that the present study represents preliminary candidate screening following LOR-family analysis, and only a limited set of physiological indices were measured to assess stress responses. Further physiological and molecular evidence, including ROS/H_2_O_2_ accumulation, electrolyte leakage, relative water content, chlorophyll fluorescence, photosynthetic performance, and the expression of antioxidant-and osmotic-response-related genes, is still required to fully elucidate the detailed regulatory mechanism of *ScLOR16*. These indicators will be prioritized in our future research to further dissect the molecular basis of *ScLOR16*-mediated stress responses. Furthermore, additional molecular-biological evidence, including protein–protein interaction assays and transcriptomic analyses, is needed to uncover the direct regulatory pathways associated with *ScLOR16*.

Previous functional studies of *AtLOR* genes have mainly focused on plant disease resistance pathways, whereas LOR genes in soybean, rapeseed, and foxtail millet have primarily been predicted to participate in drought and salt tolerance based on transcriptomic analyses, with limited functional validation in vivo. Low temperature, drought, and high salinity are among the major abiotic stresses restricting tomato growth, yield formation, and fruit quality [40,41]. The present study provides functional evidence linking *ScLOR16* to the modulation of cold-and drought-stress responses through VIGS-mediated silencing, addressing the limited understanding of LOR gene functions in abiotic stress responses of wild Solanaceae germplasm. Integrating the findings of this study, the *ScLOR* family in *S. lycopersicoides* appears to have undergone functional diversification during evolution, with different members exhibiting distinct responses to cold, drought, and salt stresses. As a potential negative regulator shared by cold and drought response pathways, *ScLOR16* may suppress antioxidant activation and proline accumulation, thereby reducing plant stress tolerance.

To date, systematic genome-wide identification and analysis of the LOR gene family in cultivated tomato (*Solanum lycopersicum*) remains unreported. Future work in our group will conduct a full genome-wide characterization of the *SlLOR* family, identify the precise *ScLOR16* orthologue in cultivated tomato, and further compare their structural features and stress regulatory functions. Subsequent gene-editing validation in cultivated tomato will clarify functional conservation or divergence between wild and cultivated LOR homologs.

The practical importance of abiotic stress related traits varies greatly depending on cultivation systems and agronomic management intensity. Traits conferring cold and drought tolerance are particularly valuable under low-input open-field conditions with frequent environmental fluctuations, whereas their relative weight declines in high-technology greenhouses where temperature and water supply can be precisely controlled.

It should be emphasized that the current results obtained based on VIGS are insufficient to establish *ScLOR16* as a practically meaningful breeding target. Although the functional characterization of this individual gene remains preliminary, the entire LOR gene family deserves further attention in research related to *Solanum lycopersicoides* improvement. Moreover, candidate genes within this family require further validation across diverse genetic backgrounds. Previous reports have demonstrated links between LOR-family members and plant defence mechanisms, and future studies should evaluate potential trade-offs among abiotic-stress responses, yield performance and disease resistance.

## 5. Conclusions

In this study, the LOR gene family was identified and systematically characterised at the genome-wide level in *Solanum lycopersicoides*. Comprehensive analyses of phylogenetic relationships, gene structures, conserved motifs, cis-acting elements, chromosomal distribution, and collinearity revealed the evolutionary characteristics of the *ScLOR* family. Expression profiling showed that *ScLOR16* was strongly induced by cold and drought stresses and exhibited distinct tissue-specific expression patterns. Subcellular localisation analysis indicated that ScLOR16 was localised to both the nucleus and cytoplasm. Furthermore, VIGS-mediated functional biological analyses revealed that *ScLOR16*-silenced plants exhibited enhanced stress tolerance under cold and drought conditions, accompanied by higher activities of antioxidant enzymes (SOD and POD), greater proline accumulation, and lower TBARS levels. Collectively, these results indicate that *ScLOR16* may participate in cold and drought stress responses in *Solanum lycopersicoides*, and provide fundamental evidence for future in-depth investigation of stress-response mechanisms in this species.

## Figures and Tables

**Table 1:** Analysis of LOR Protein Characteristics in *S. lycopersicoides*.

Gene Name	Gene ID	Chr Location	Number of Amino Acids	Theoretical pI	Molecular Weight (Da)	Instability Index	Aliphatic Index	Subcellular Localization Prediction
ScLOR1	Scoides03g359100.1	Chr3	211	5.18	23,596.63	37.09	80.71	Nucleus
ScLOR2	Scoides06g022000.1	Chr6	200	8.63	22,931.27	29.53	78.45	Golgi, Cytoplasm
ScLOR3	Scoides06g223100.1	Chr6	201	9.10	23,066.86	25.24	101.24	Cytoplasm
ScLOR4	Scoides08g017800.1	Chr8	230	9.55	25,813.89	41.26	90.61	Chloroplast, Cytoplasm
ScLOR5	Scoides08g027100.1	Chr8	212	9.55	24,127.73	42.79	91.32	Chloroplast, Mitochondrion
ScLOR6	Scoides08g027200.1	Chr8	205	9.46	23,546.11	40.86	92.98	Chloroplast
ScLOR7	Scoides08g027300.1	Chr8	212	9.35	24,114.81	42.59	100.05	Chloroplast, Mitochondrion
ScLOR8	Scoides08g027400.1	Chr8	173	9.19	19,624.71	30.92	99.48	Chloroplast
ScLOR9	Scoides08g027500.1	Chr8	198	9.25	22,356.79	36.85	77.63	Chloroplast, Nucleus
ScLOR10	Scoides08g246000.1	Chr8	208	8.50	23,393.78	45.91	82.79	Golgi, Chloroplast, Nucleus, Extracellular
ScLOR11	Scoides09g246400.1	Chr9	203	6.95	23,022.41	41.53	85.91	Nucleus, Extracellular
ScLOR12	Scoides09g246600.1	Chr9	216	9.08	23,915.65	30.45	90.32	Nucleus, Cytoplasm
ScLOR13	Scoides09g246800.1	Chr9	225	8.64	24,739.54	25.91	90.22	Nucleus, PlasmaMembrane
ScLOR14	Scoides09g246900.1	Chr9	195	6.32	21,822.20	33.05	98.87	Golgi, PlasmaMembrane, E.R.
ScLOR15	Scoides09g319000.1	Chr9	266	9.94	30,220.57	51.21	67.78	Nucleus, Chloroplast, Mitochondrion
ScLOR16	Scoides10g062800.1	Chr10	214	8.41	23,993.47	46.96	87.85	Chloroplast, Vacuole
ScLOR17	Scoides12g068300.1	Chr12	197	9.46	22,463.18	34.27	89.95	Chloroplast, Mitochondrion
ScLOR18	Scoides12g068400.1	Chr12	203	9.20	22,781.13	36.47	85.32	Chloroplast
ScLOR19	ScoidesUng197900.1	ChrUn	202	9.06	22,505.06	30.28	92.48	Cytoplasm

## Data Availability

The original contributions presented in this study are included in the article/Supplementary Material. Further inquiries can be directed to the corresponding authors.

## Supplementary Materials

The following supporting information can be downloaded at: https://www.mdpi.com/article/10.3390/biology15171542/s1, Table S1: All primers used in this study; Table S2: *ScLOR16* silencing fragment; Table S3: All protein sequences used in this study; Table S4: Cis-acting elements in the promoters of *ScLOR* gene family; Table S5: RT-qPCR data under cold, drought and salt stresses; Table S6: Relative expression levels of *ScLOR16* in different tissues; Table S7: Detection of gene silencing efficiency of *ScLOR16*; Table S8: Stability validation of reference genes.

## Abbreviations

The following abbreviations are used in this manuscript:

LOR	*LURP*-one related
VIGS	Virus-Induced Gene Silencing
SOD	Superoxide Dismutase
POD	Peroxidase
TBARS	Thiobarbituric-acid-reactive substances
Pro	Proline
ABA	Abscisic Acid
ROS	Reactive Oxygen Species
SA	Salicylic Acid
CDS	Coding Sequence
